# Systematic Investigation on the Glass Transition Temperature of Binary and Ternary Sugar Mixtures and the Applicability of Gordon–Taylor and Couchman–Karasz Equation

**DOI:** 10.3390/foods11121679

**Published:** 2022-06-07

**Authors:** Martin Schugmann, Petra Foerst

**Affiliations:** Institute of Process Systems Engineering, Technical University of Munich, Gregor-Mendel-Straße 4, 85354 Freising, Germany; martin.schugmann@tum.de

**Keywords:** amorphous state, glass transition, carbohydrate mixtures, sugar mixtures, DSC, amorphization, molecular weight, food polymer, Gordon–Taylor equation, Couchman–Karasz equation

## Abstract

Glass transition temperatures (*T_g_*) of carbohydrate mixtures consisting of only one monomer and glycosidic binding type (aldohexose glucose, α1-4-glycosidic bonded) were studied by differential scanning calorimetry (DSC). The aim of this work was to systematically assess the predictability of *T_g_* of anhydrous binary and ternary sugar mixtures focusing on the components *T_g_*, molecular chain length, and shape. Binary systems were investigated with glucose as a monosaccharide and its linear di-, tri-, tetra-, penta-, hexa-, and heptasaccharides. Additionally, the *T_g_* of ternary carbohydrate systems prepared with different glucose/maltose/maltotriose mass fractions were studied to evaluate the behavior of more complex mixtures. An experimental method to prepare fully amorphized, anhydrous mixtures were developed which allows the analysis of mixtures with strongly different thermodynamic pure-component properties (*T_g_*, melting temperature, and degradation). The mixtures’ *T_g_* is systematically underestimated by means of the Couchman–Karasz model. A systematic, sigmoidal deviation behavior from the Gordon–Taylor model could be found, which we concluded is specific for the investigated glucopolymer mixtures. At low concentrations of small molecules, the model underestimates *T_g_*, meeting the experimental values at about equimolarity, and overestimates *T_g_* at higher concentrations. These deviations become more pronounced with increasing *T_g_* differences and were explained by a polymer mixture-specific, nonlinear plasticizing/thermal volume expansion effect.

## 1. Introduction

Sugars and carbohydrate matrices are used as sweeteners, to preserve foods, as fillers for tablets, as coatings for tablets or candies, or they fulfill a protective function for sensitive biomolecules from environmental influences [1,2]. In particular, the glass transition temperature (*T_g_*) plays a central role during manufacturing processes, post-production stability, or shelf life of sugar-rich, amorphous goods in pharmaceutical and food applications [3,4,5,6]. If *T_g_* is exceeded, strong property changes occur. The viscosity of the structure decreases abruptly during the transition from the glassy to the rubbery state, and at the same time, the molecular mobility increases. Problems that can occur are recrystallization, caking, or agglomeration [7,8,9,10]. A well-elaborated example with great industrial importance is the caking phenomenon of lactose-rich dairy powders. In this case, the exceedance of *T_g_* can lead to caking of the product either by the formation of sinter bridges between the particles during storage due to the decreasing viscosity of the substance or due to recrystallization [11]. In this context, the *T_g_*-related kinetics of recrystallization was investigated by Ibach et al. [12,13]. In addition, wall deposition during spray drying is an important issue during the processing of food and pharmaceuticals and the sticky point temperature is a function of *T_g_* [14,15].

In many products, sugars occur as mixtures; e.g., maltodextrins, molasses, fruit juices, or honey. For product safety and product quality, it is important to be able to predict the *T_g_* of sugar mixtures as accurately as possible. The knowledge about the glass transition behavior is relevant, on the one hand, as it allows materials to be assessed in advance with regard to their usability, and on the other hand, the glass transition temperature can be adapted to require or given storage and processing conditions by means of appropriate additive addition. In this context, high molecular weight excipients such as maltodextrins are often added to increase *T_g_*. There are a number of models that can be used to mathematically describe glass transition temperatures of component mixtures. The Gordon–Taylor equation (Equation (1)) or the Couchman–Karasz equation (Equation (2)) are usually used for the prediction [16,17]. These equations are based on the free volume theory and additivity of basic thermophysical properties. For the former, the glass transition temperatures *T*_*g*1_ and *T*_*g*2_ of the pure substances, their mass fractions *w*_1_ and *w*_2_, and a curvature coefficient *K* are required, which is fitted from several measured values of *T_g_* with varying compositions:

(1) $$T_{g}=\frac{w_{1}*T_{g1}+K*w_{2}*T_{g2}}{w_{1}+K*\;w_{2}}$$

In a limited concentration range, the *T_g_* of binary mixtures, e.g., consisting of a sugar and water, is usually sufficiently well expressed by the Gordon–Taylor equation. This empirical estimation of the glass transition is therefore widely used in industry and research. To describe systems of more than two components with the help of the Gordon–Taylor equation, the total solid mass can be taken as one component and the water contained as the second component. For instance, Arvanitoyannis et al. employed this approach for glucose–fructose–water mixtures [18].

The Couchman–Karasz equation requires the glass transition temperatures *T_g,i_* of the components, the changes in specific heat capacities Δ*c_p,i_* at glass transition, and the mole fractions *x_i_* of the pure substances which in principle make it also a simple tool for estimating *T_g_* of a mixture:

(2) $$T_{g}=\frac{\sum_{i=1}^{n}\left(x_{i}*\Delta c_{p,i}*T_{g,i}\right)}{\sum_{i=1}^{n}\left(x_{i}*\Delta c_{p,i}\right)}$$

Both the Gordon–Taylor and Couchman–Karasz equations are based on the free volume theory and apply under the assumption that the mixing partners are similar in shape and size and are ideally mixed. This means that the free volume of the substances behaves additively and no interactions occur between the mixing partners. However, it is difficult to predict the *T_g_* of sugar mixtures exactly because the mixing behavior might be nonlinear [19]. This was also illustrated by the example of selected binary mixtures of glucose, galactose, and maltotriose [20]. Additionally, for molasses, the glass transition temperature cannot be predicted from the *T_g_* of its main components sucrose, glucose, and fructose (together 89–97% of the mass) [21]. Up to now, almost no systematic studies have been carried out on the influence of the mixture composition on the glass transition of anhydrous sugar mixtures, especially on the systematic variation of molecular size. Seo et al. found for mixtures of mono-, di-, and trisaccharides (sorbitol, glucose, sucrose, trehalose, maltotriose) that the investigated mixtures of monosaccharide–monosaccharide and disaccharide–disaccharide could be described by the Gordon–Taylor equation, but not mixtures of monosaccharide–disaccharide and monosaccharide–trisaccharide. They concluded that the size and shape of the sugars play an important role with respect to the glass transition temperature of the mixtures [22]. The main finding of these studies was that it is difficult to make a precise prediction. Generally valid relationships could not be derived so far. It is not yet possible to predict the glass transition behavior for random sugar mixtures [23,24,25,26].

This study aims to make a contribution in this direction and systematically investigates a homologous series of sugar mixtures with one monosaccharide and focuses on the variation of molecular size of the mixing partner with the same monomer. This minimizes the influence of different monomer shapes/side chains and gives insight into the substance group-specific glass transition behavior.

Glucose with glucose homopolymers linked in the same way was used as a model sugar system. Using the selected systems, the composition-dependent glass transition behavior, as well as the predictability of mixture glass transition temperatures by means of the above equations, is investigated by comparison with values measured in DSC.

A major experimental problem here is that sugars are extremely hygroscopic in the amorphous state, water acts as a plasticizer and greatly lowers the glass transition temperature [8]. Complete removal of water from the sugars prior to measurement is therefore inevitable and at the same time difficult, and different methods with specific advantages and disadvantages are available for water removal. The single components of carbohydrate mixtures usually have different thermodynamic properties regarding melting, glass transition, and degradation behavior. Therefore there is a need for a sugar-specific suitable methodology. This study also faces this problem. A combined method of freeze-drying, thermogravimetry, and in situ thermal treatment in DSC is presented, which enables residual water removal and full amorphization while minimizing thermal stress.

## 2. Materials and Methods

### 2.1. Materials

Materials used in this study are listed below in Table 1:

### 2.2. Mixture Preparation

**Figure 1:**
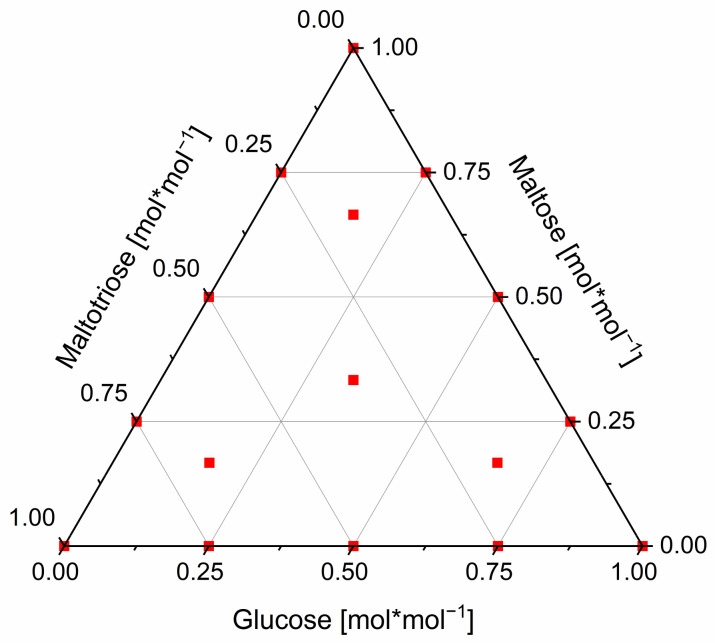
Simplex experimental design of the investigated ternary glucose–maltose–maltotriose mixtures.

Six binary mixtures of glucose with the mixing partners maltose, maltotriose, maltotetraose, maltopentaose, maltohexaose, and maltoheptaose were produced. The molar fraction of glucose in the mixtures was varied between 0% and 12.5%, 25%, 50%, 75%, 87.5%, and 100%. All molar fractions refer to the total carbohydrate mass of the mixture. Furthermore, a modified simplex axial (centroid) mixing plan with the grade 3 was created (extended by the molar fractions of 25–75% on the simplex edges) to systematically investigate ternary mixtures of glucose, maltose, and maltotriose. The ternary experimental space is plotted in Figure 1:

The sugars were weighed out to a total sugar mass of 0.2 g according to the desired molar ratios, dissolved in 5 mL MilliQ water (MilliQ IQ Water Purification System; Merck KGaA, Darmstadt, Germany), and then allowed to stand for 24 h to dissolve completely. For accurate dosing of smaller amounts of saccharides, a larger amount was dissolved in MilliQ water in a defined ratio and dosed accordingly. Subsequently, the solutions were transferred to flat aluminum sample trays (filling height about 4 mm, ROTILABO 20 mL; Carl Roth GmbH + Co. KG; Karlsruhe; Germany), shock frozen with liquid nitrogen, and freeze-dried at 10 Pa for 48 h (Freeze Dryer Alpha 2-4 LSCPlus; Martin Christ Gefriertrocknungsanlagen GmbH; Osterode am Harz; Germany). Subsequent post-drying was performed at 1 Pa and 25 °C for 72 h. The lyophilized samples were stored in vacuum desiccators over P_2_O_5_ for at least two weeks until further use.

### 2.3. Measurement Methodology

Two methods are available for the preparation of amorphous, anhydrous carbohydrates: shock melt quenching and freeze-drying [27,28,29,30]. Shock melt quenching is understood to be the melting of crystalline substances followed by rapid cooling to prevent crystallization before the glass transition. In this study, a combination of freeze-drying and in situ thermal treatment (for residual dehumidification) before the actual measurement in the DSC was used for the preparation of a completely dried sample. This combines the specific advantages of freeze-drying and shock melt quenching. The actual drying was carried out by freeze-drying, while the residual dehumidification was carried out in the DSC at elevated temperatures. This procedure allows an ideal mixing of the components, effective and controlled removal of water (especially hydrate water in the case of maltose monohydrate) without thermal stress (degradation), and the need of handling the sample at ambient atmospheric conditions after dehumidification and the monitoring of possible crystallization.

**Figure 2:**
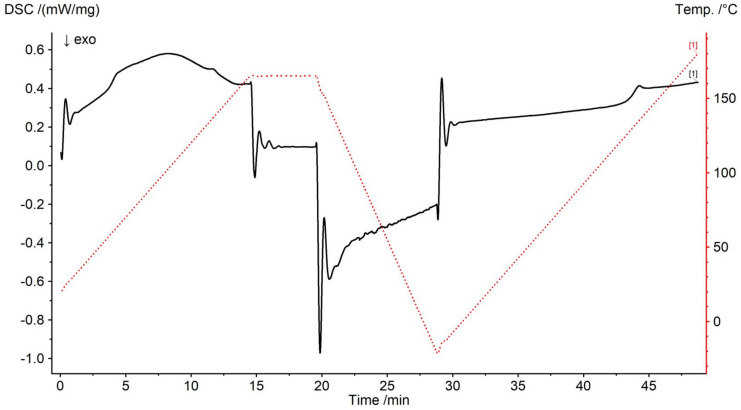
Thermogram of simultaneous residual dehydration and subsequent determination of the glass transition of maltotriose. The endothermic profile of the signal, which shows the release of water, can be clearly seen as a hump during the first heating phase.

To measure the glass transition temperatures in the DSC, 4 mg ± 0.2 mg of the sample from each mixture generated was weighed in a DSC aluminum crucible (Concavus 30 mL; NETZSCH-Gerätebau GmbH; Selb; Germany) with a precision balance (Shimadzu AUW220D; Shimadzu Germany GmbH; Duisburg; Germany; repeatability = 0.02 mg) under a dry atmosphere in a dry box (Captair pyramid; Erlab DFS S.A.S.; Val de Reuil Cedex; France) and then directly transferred to the DSC (Netzsch 214 Polyma; NETZSCH-Gerätebau GmbH; Selb; Germany). Figure 2 shows an exemplary DSC thermogram of the heating and cooling steps with the associated residual water removal and subsequent measurement of the glass transition of pure maltotriose.

**Figure A1:**
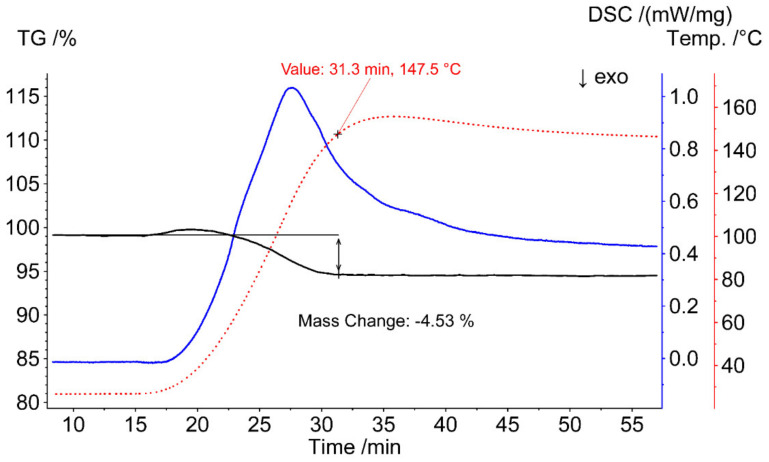
Exemplary simultaneous thermogravimetry and differential thermal analysis thermogram of a maltotriose sample with relatively high water content indicating a complete water release of 4.5% at about 148 °C.

Prior to the actual measurement step, the sample was equilibrated in the DSC at 20 °C for 5 min (not shown in Figure 2). To remove any residual water which could not be removed by freeze-drying and storage, a dynamic step was added to the DSC measurement program. Carbohydrates were therefore heated to 165 °C at a rate of 10 °C/min and held at this temperature for 5 min to dry them completely. Besides drying and the possibility to observe the completeness of water removal, the heating of the substance above the glass transition fulfills the purpose of erasing its thermal history from the physical aging. The samples were then cooled to −20 °C at a rate of 20 °C/min. Finally, a second heating phase was performed at 10 °C/min to 180 °C. The glass transition was detected in this last step as an endothermic change in heat flux (see Figure 2). The values of the glass transition temperatures (*T_g_*) as well as the change of the specific heat capacity at the glass transition (Δ*c_p_*) were determined from DSC thermograms as the midpoint between the initial and the final point in the heat flux-temperature diagram using Netzsch Proteus V7.0 software in compliance with ISO 11357-2. The crucibles were measured open, i.e., without lids, so that the water in the sample could escape completely. An empty aluminum crucible served as a reference for baseline calibration. All experiments were performed under an inert atmosphere with nitrogen (high purity, grade 5.0) at a flow rate of 60 mL/min. Experiments with pure components were repeated at least five times and measurements of mixtures were performed in triplicate. The order in which experiments were performed was randomized. Complete water release was confirmed using simultaneous thermogravimetry differential thermal analysis (STA; Netzsch 449 F3 Jupiter; NETZSCH-Gerätebau GmbH; Selb; Germany) with an analog temperature program (see Figure A1 in Appendix A). DSC and STA were temperature and sensitivity calibrated for the specific heating rate, gas flow, and crucible selection using six ultra-pure standards for thermal analysis (substances: Adamantane/C_10_H_16_, In, Sn, Bi, Zn, and CsCl; purity > 99.999%; in accordance with ASTM E 967). For determination of heat capacity, the DSC was also calibrated according to ASTM E 1269 (baseline aluminum crucible and sapphire standard). Mass-related quantities such as Δ*c_p_* were corrected for the fraction of water loss.

## 3. Results and Discussion

### 3.1. Calorimetric Results during Sample Preparation

For all investigated samples, a broad endothermal hump/peak (water release) was observed in the range of 60 and 140 °C during the first heating period of DSC treatment. The hump is associated with the evaporation of residual water. The hump/peak ended in a constant line, which indicated the end of water removal. The temperature range of the water release coincides with selective, extended thermogravimetric investigations of the STA and was therefore considered to indicate a complete residual water removal.

In this context, Figure A1 in Appendix A exemplarily shows the thermogram of a maltotriose sample with relatively high water content (analyzed after freeze-drying). The end of mass loss during the heating phase at about 148 °C indicates that the water has been completely released even for samples where it can be assumed that the water release takes longer due to higher water contents. Furthermore, during DSC treatment no exothermal effects could be observed, which showed that no crystallization occurred during measurement.

### 3.2. Thermoanalytic Properties of Pure Components

Table 2 lists the thermoanalytically obtained data of the pure substances used in this study.

With increasing molecular weight, the glass transition temperature also increases from 38.87 °C for glucose to 188.44 °C for maltoheptaose. This is consistent with the findings for maltodextrins of various dextrose equivalents by Saavedra-Leos et al., Roos et al., and Mayhew et al. [24,31,32].

However, the increase in our study is not linear. The *T_g_* midpoint temperatures measured in this study are in good agreement with already known literature values for the carbohydrates glucose, maltose, maltotriose, and maltohexaose [20,22,27,28,33]. Small differences between published *T_g_*-values of the same substance can be attributed to different amorphization methods applied (see the previous chapter), possibly existing residual water contents and different heating rates used. Furthermore, the value of the change in specific heat capacity decreases with the number of monomers in the molecule. This general trend is also consistent with the reported values for structurally more undefined maltodextrins, which decreased with increasing molecular weight [24]. Nevertheless, values for Δ*c_p_* at the glass transition for pure or defined carbohydrates are comparatively scarce and literature values for glucose range from 0.63 to 0.88 J∗g^−1^∗K^−1^ and 0.61 to 0.79 J∗g^−1^∗K^−1^ for maltose, respectively [20,27].

### 3.3. Binary Glucose–Glucopolymer Mixtures

**Figure 3:**
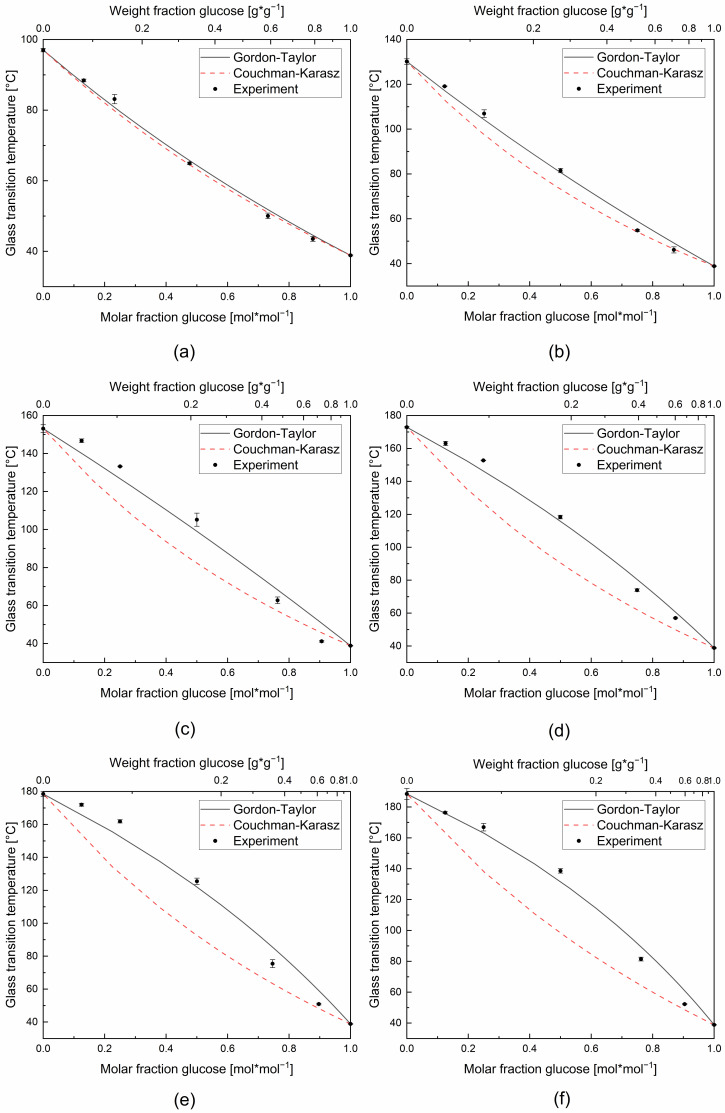
Experimental glass transition temperatures, Gordon–Taylor fit and Couchman–Karasz curve for the investigated, anhydrous binary glucose mixtures with (**a**) maltose, (**b**) maltotriose, (**c**) maltotetraose, (**d**) maltopentaose, (**e**) maltohexaose, and (**f**) maltoheptaose.

**Figure 4:**
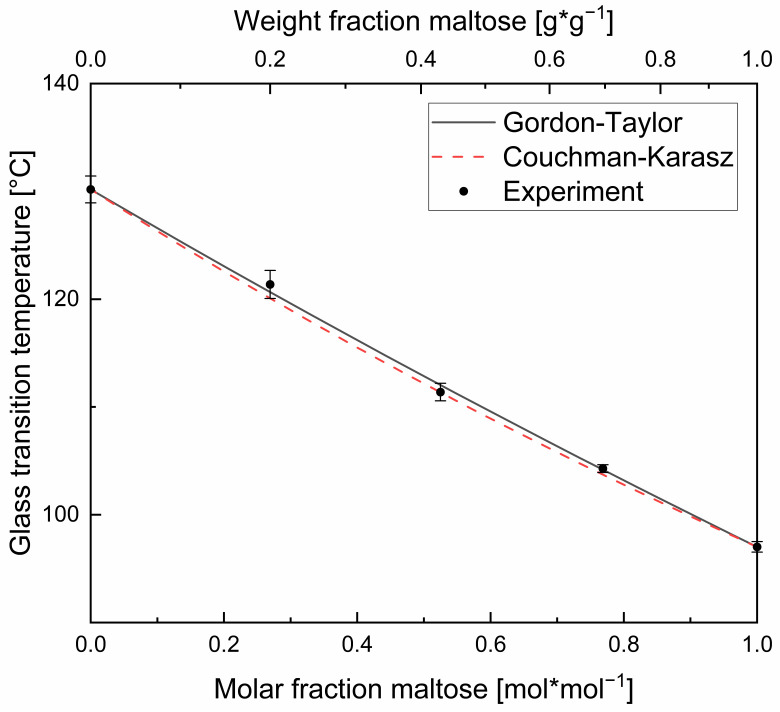
Experimental glass transition temperatures, Gordon–Taylor fit and Couchman–Karasz curve for the investigated, anhydrous binary maltose–maltotriose mixture.

For all binary and ternary systems, only one well-defined glass transition temperature was observed. This fact confirms good miscibility and compatibility between glucose and the higher molecular weight components used in this study [28,34,35,36]. The experimental and predicted values for *T_g_* using the Gordon–Taylor model (Equation (1)) and the Couchman–Karasz model (Equation (2)), respectively, as a function of the solid molar fraction of the pure components are shown in the following Figure 3 and Figure 4.

**Figure 5:**
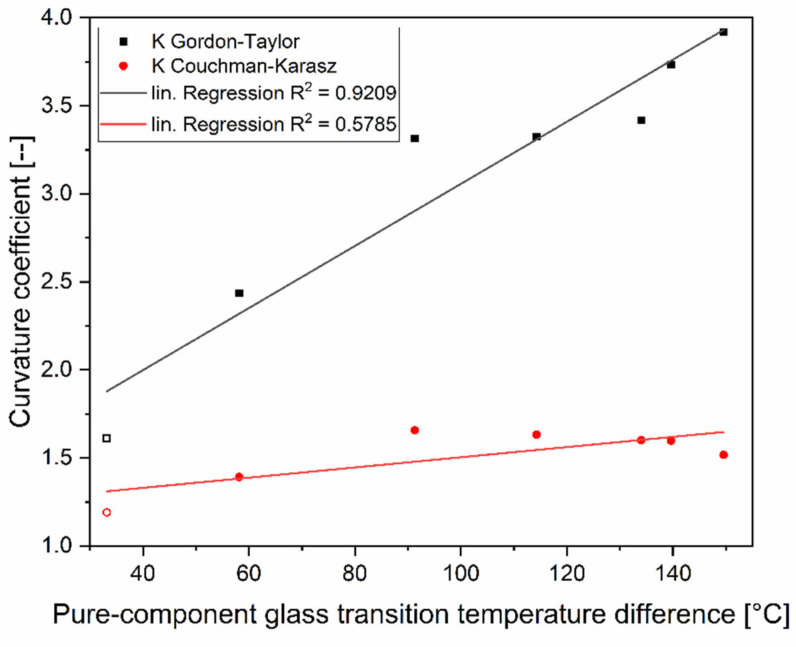
Empirical curvature coefficients *K* (Gordon–Taylor fit) and theoretical *K*-values (derived from the pure-component Δ*c_p_* values) as a function of the pure-component glass transition temperature difference Δ*T_g_* for the binary carbohydrate mixtures used in this study. Also included are the values of the binary maltose–maltotriose mixture from the ternary mixture design of this study (symbols without filling).

Plasticizers in amorphous polymers decrease the glass transition temperature of their mixture. The expected general decrease of *T_g_* when the glucose molar fraction was increased can be clearly observed for all the sugar samples. The monosaccharide glucose can be considered here as a plasticizer. The curvature coefficients (*K*-values) were fitted with the Excel 2016-Solver and the GRG nonlinear algorithm. These obtained empirical *K*-values, quantifying the plasticization rate in the samples ranged from 2.43 to 3.92 for the glucose mixtures and was 1.61 for the binary maltose–maltotriose mixture of the ternary mixture design (edge of the simplex). The values are displayed in Figure 5. From Figure 3 and Figure 4, it can be seen that the graphical relationship between glass transition temperature and plasticizer content (molar basis) turns from concave to increasingly convex with rising *T_g_* difference (Δ*T_g_*) of the mixing partners, whereas *K*-values above unity indicates that the graphical relationship on weight fraction basis was concave in all the cases [37].

The theoretical *K*-values, obtained using the change in heat capacity on the glass transition of the pure components (Δ*c_p_*), were much lower than the empirical *K*-values, see also Figure 5. This leads to considerately bad predictions by the Couchman–Karasz model, which is illustrated in Figure 3 and Figure 4 by large differences between the measured and calculated values. From these figures, it can also be observed that the Couchman–Karasz model deviates more from the experimental values than the Gordon–Taylor model. Moreover, a clear trend of rising deviation with the increase of the saccharides’ molar mass difference was found between the Couchman–Karasz model and the measured values.

As mentioned before, the Δ*c_p_* values of glucopolymers decreased with increasing molecular weight (see Table 1). This should lead to increasing theoretical *K*-values for binary glucose mixtures with glucopolymers of increasing molecular weight or glass transition temperature, respectively, since the *K*-value is formed as the quotient of the Δ*c_p_* of the glucose and the Δ*c_p_* of the polysaccharide. In Figure 5, an upward trend is clearly observable in the empirical (Gordon–Taylor fitted) *K*-values but is much less pronounced for the theoretical *K*-values (Couchman–Karasz). Also included in the plot is the calculated *K*-value of the binary maltose–maltotriose mixture from the ternary mixture design of this study (symbols without filling). A linear relationship between *K*-value and Δ*T_g_* seems to explain the empirical *K*-value well with a coefficient of determination of R^2^ = 0.9209 whereas the predictability of the theoretical *K* is poorer with R^2^ = 0.5785.

The root-mean-square deviation (*RMSD*; Equation (3)) is a frequently used measure of the differences between observed values and the values predicted by a model. The normalized root-mean-square deviation (*nRMSD*; Equation (4)) in turn can be used as a scale-independent measure of the deviation between experimentally gathered *T_g_*-values (*T_g,i_*) and values predicted by the nonlinear Gordon–Taylor model (*T_gGT,i_*). It is defined as the quotient of the root-mean-square deviation and the arithmetic mean of measured glass transition temperatures of a mixing row with *n* data points:

(3) $$RMSD=\sqrt{\frac{\sum_{i=1}^{n}{\left(T_{g,i}-T_{gGT,i}\right)}^{2}}{n}}$$

and

(4) $$nRMSD=\frac{RMSD}{\frac{1}{n}\sum_{i=1}^{n}T_{g,i}}$$

**Figure 6:**
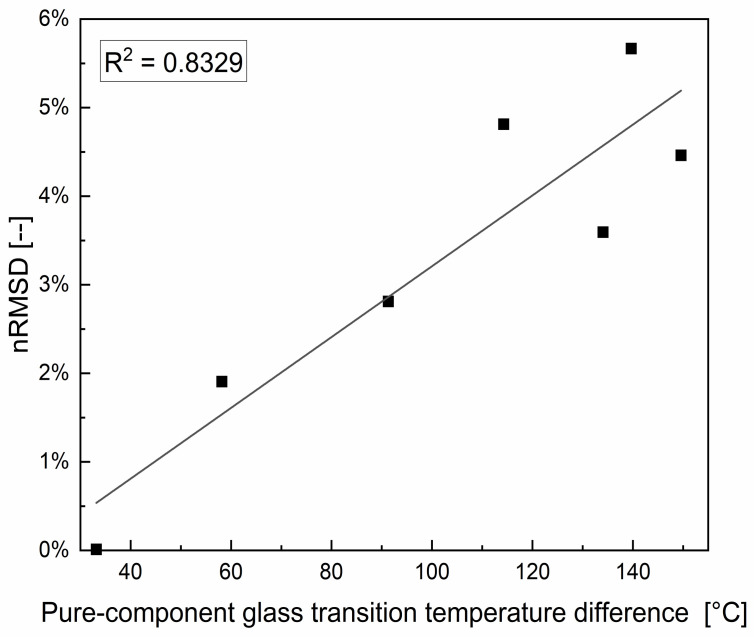
The normalized root-mean-square deviation (*nRMSD*) as a scale-independent measure of the deviation plotted against the difference of pure-component glass transition temperatures Δ*T_g_* of the investigated binary mixtures.

The plot of the *nMRSD* against the difference of pure-component glass transition temperatures Δ*T_g_* is shown in Figure 6. Mixtures with small Δ*T_g_* differences like glucose–maltose (Δ*T_g_* = 58.2 °C) are better described by the Gordon–Taylor equation than mixtures with rising differences in glass transition. This is reflected in the tendency of the increasing *nRMSD* for rising Δ*T_g_* as a normalized measure for differences between the experimental and the predicted values by the Gordon–Taylor model. The observation is further supported by the glass transition curve of maltose and maltotriose from the ternary mixture design. Here, Δ*T_g_* is only 33.2 °C and at the same time, the highest accuracy for the Gordon–Taylor model is found with an *nRMSD* of 0.43. Therefore, we believe that already the molecular size difference plays a critical role in the predictability of the glass transition temperature of the mixture.

A closer look at the experimental data in Figure 3 and Figure 4 further reveals that the measured *T_g_* slightly deviates from the Gordon–Taylor model in a systematic manner. At high molar fractions of glucose, the model overestimates the experimental glass transition temperatures of all binary systems investigated in this study, and at lower fractions, they are underestimated. The experimental values intersect the Gordon–Taylor function in all mixture series at approximately equimolarity. This also applies to the disaccharide–trisaccharide mixture of maltose and maltotriose. On the one hand, a similar deviation behavior from the Gordon–Taylor equation is noticeable in the case of the here investigated mixtures of glucose and its linear di-, tri-, tetra-, penta-, hexa-, and heptasaccharides. On the other hand, the percentage deviation becomes larger with increasing *T_g_* or size difference of the mixtures partners, evident in the increasing trend of the *nRMSD*. Together, we conclude that a strengthening of the sigmoidal deviation effect appears with increasing molecular size difference for glucose–glucopolymer mixtures. For mixtures of monosaccharides and disaccharides, namely sorbitol–sucrose and glucose–sucrose, Seo et al. found a similar sigmoidal deviation of the experimental values from the Gordon–Taylor curvature behavior: the binary mixture series of sorbitol-sucrose showed a deviation with the same upward-downward systematic as for the mixtures of our study, whereas for the glucose–sucrose mixture the deviation was reversed. In the latter case, the Gordon–Taylor model underestimates the experimental data at high glucose levels [22]. As sucrose was the common component in this mixture and the molecular weight of sorbitol and glucose are very similar, the molecular shape seems to play a critical role in the glass transition. The sugars of our study all had the same monosaccharide unit and glycosidic bonding type. Therefore, we propose that the glass transition temperatures of sugar mixtures consisting of sugar units with different sizes but the same monomer shape (and glycosidic bonding) will deviate from the Gordon–Taylor equation but in a systematic and predictable way regarding, curvature, intersection with the model and extent of the deviation.

The Gordon–Taylor and the Couchman–Karasz models are proposed based on the ideal volume of mixing (additivity of free component volumes) and the linear change in volume with temperature. From the systematic deviation in the same direction of all glucose–glucopolymer mixing partners investigated in this study, we conclude that the volume of mixing deviates from ideal behavior, and there is generally a similarity for the nonlinearity from ideal volume mixing for (comparable molar ratios) of this group of substances. Additionally, the good agreement with the Gordon–Taylor equation at equimolarity and therefore the ideal free volume additivity at this point seems to be specific. These findings could possibly be extended to a broader group of substances, supported by further investigations with glucopolymers (e.g., same shape, different glycosidic bindings, or intramolecular branchings). In this context, it is interesting to remark on the findings of Saavedra-Leos et al. in their study of mixtures containing maltodextrins. The authors showed that differences in branching and therefore differences in the functional groups available for establishing intra- and inter-molecular interactions have an important influence on the glass transition behavior of the mixture [38].

### 3.4. Ternary Mixtures

**Figure 7:**
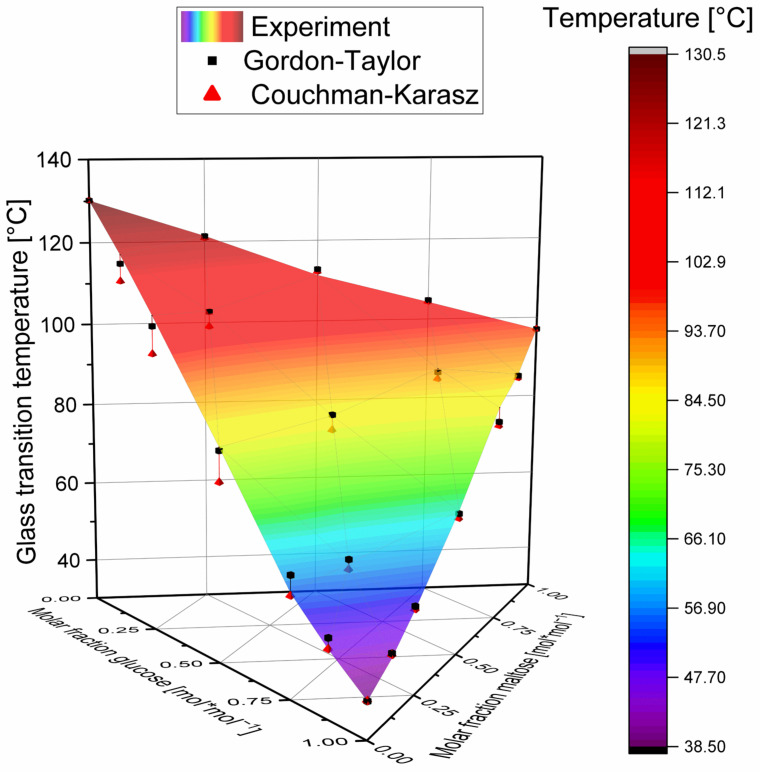
Experimental glass transition temperatures (color grade) and glass transition temperatures according to the Gordon–Taylor (black cubes) and Couchman–Karasz equations (red tetrahedrons) of the ternary glucose–maltose–maltotriose mixtures as a function of the molar fraction. For the equimolar mixture (simplex center point), the glass transition temperature of the Gordon–Taylor equation corresponds to the mean value of the three fits with glucose, maltose, or maltotriose as the first component and the mixture of the remaining sugars as the second component.

In the course of this study, ternary mixtures of glucose, maltose, and maltotriose were also investigated. The experimental result is shown graphically in Figure 7 as a color map in the ternary surface diagram. (Furthermore, Table A1 in Appendix A lists the molar composition of the mixtures, the measured glass transition temperatures, and the values predicted according to the Gordon–Taylor and Couchman–Karasz models.)

It can be seen that the measured glass transition temperatures of the ternary mixtures are all higher than the predicted values of the Couchman–Karasz model (red tetrahedrons), which is in accordance with the findings of the binary mixture systems investigated in this study. Additionally, the difference between Couchman–Karasz model and measurement is larger with an increasing proportion of maltose and/or maltotriose. This observation is consistent with the results of the binary mixtures investigated, where within such a series of mixtures the distance between the Couchman–Karasz model and the experimental values is also higher for large proportions of the higher molecular weight substance. It can thus be assumed that the deviation system already discussed for the unbranched α1-4-bonded glucopolymers can also be extended to more complex systems from this substance class. In the following, the ternary system is analyzed in more detail using an approach that interprets the results in a binary manner.

**Figure 8:**
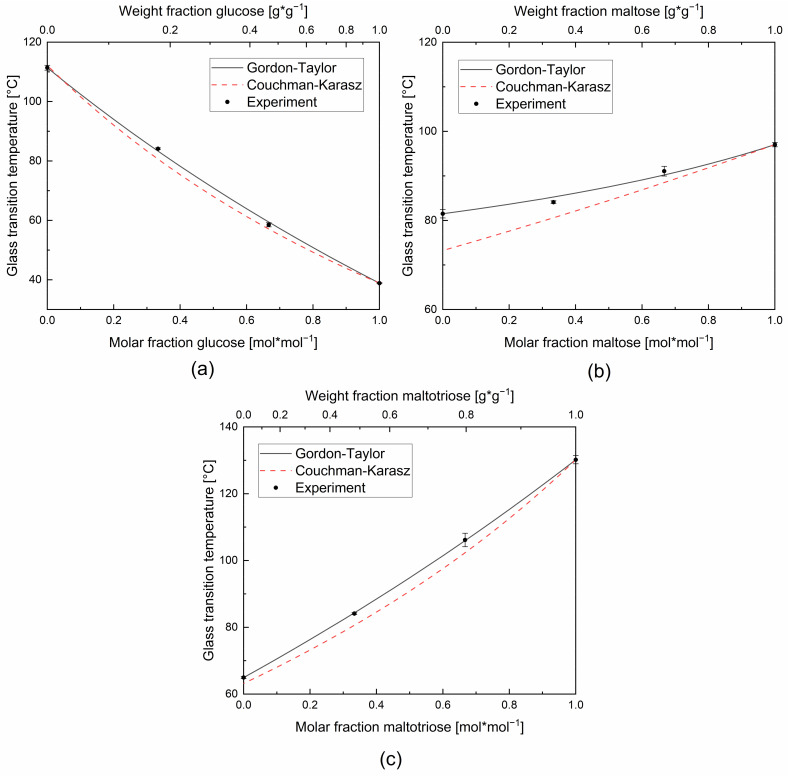
Experimental glass transition temperatures and glass transition temperatures according to the Gordon–Taylor and Couchman–Karasz equations of the ternary glucose–maltose–maltotriose mixtures as a function of molar fraction and weight fraction of the main sugar (**a**) glucose, (**b**) maltose, and (**c**) maltotriose (axis labels). As the proportion of the main sugar varies for each composition, the remaining sugars are mixed equimolar.

In order to describe systems of more than two components with the help of the Gordon–Taylor equation, one sugar was taken as the first component and the mixture of the remaining sugars as the second component. The series of mixtures were considered in such a way that this second component was always equimolar among itself (three bisecting, straight lines of the simplex). This binary expression of the ternary mixtures is plotted in Figure 8.

It can be seen that the Couchman–Karasz model again deviates strongly from the experimental values, especially for the variation of the molar fraction of maltose and maltotriose (see Figure 8b,c, respectively). A consistent deviation systematic of the measured values from the Gordon–Taylor fit can be observed here as well, which cannot be described by the model’s curvature coefficient *K*. The experimental values exceed the model prediction with a low fraction of low-molecular weight sugar, meet the prediction at about equimolarity and underrun it at higher quantities. Thus the findings are similar to the previously mentioned sigmoidal curvature deviation of the investigated binary systems. For the mixture with varying glucose content and an equimolar mixture of the remainder, the curvature behavior with respect to the Gordon–Taylor fit is the same as for the binary mixtures investigated in the previous chapter. For the other two mixing systems (maltose and maltotriose in varying molar ratios), it can be observed that the Gordon–Taylor model underestimates the measured values for a high proportion of longer-chain molecules and underestimates the measured values for a high proportion of short-chain molecules, which is in agreement with the previous findings. In conclusion, it can be concluded that, comparable with the binary systems, the difference in the pure-component glass transition temperatures of the mixture partners decides the amount of the deviation, but here in a proportional manner.

## 4. Conclusions

It was possible to analyze the glass transition of binary and ternary sugar mixtures with wide differences in pure-component *T_g_* by means of a method combining freeze-drying and in situ DSC thermal treatment. The Couchman–Karasz model on the basis of the change of heat capacity at glass transition Δ*c_p_* and the pure-component glass transition temperatures *T_g_* of the mixing partners shows very high deviations from the experimental values and consequently underestimates the mixtures’ glass transition. The glass transition temperature difference of the pure components (Δ*T_g_*) correlates well with the determined curvature coefficient *K* of the Gordon–Taylor model and a linear, rising trend of *K* was observed with Δ*T_g_*. With higher Δ*T_g_*, the predictability/quality of the Gordon–Taylor model also decreases. This leads to the conclusion that the prediction for anhydrous, more complex mixtures cannot be predicted in a blanket manner solely on the basis of composition and the thermodynamic properties of the pure components. A systematic and sigmoidal curvation behavior, which cannot be described by the Gordon–Taylor constant *K*, was found for all binary glucose–glucopolymer mixtures. The experimental values always exceed the model prediction with a low fraction of low-molecular weight sugar, meet the prediction at about equimolarity, and underrun it at higher quantities. This also applies to the binary consideration of the ternary systems in that substance group and the amount of deviation correlates well with Δ*T_g_*. These systematic deviation trends were explained with a similar, molar ratio-specific deviation from the ideal volume mixing for systems in the substance group of glucose homopolymers. As molecular shape was only varied by the chain length and not the form of a monomer, molecular backbone, or side groups, we conclude that the *T_g_* of sugar mixtures is strongly affected already by the size/aspect ratio, presumably by the uneven distribution of free volume over the entire mixing range. We propose that the deviation systematic of glass transition temperatures from the Gordon–Taylor equation is the same for sugar molecules of mixtures differing only in the chain length.

In the literature diagrams of the glass, transitions are often constructed for a wide temperature interval extending from little experimental data. This practice, as demonstrated herein, is likely not valid for precise prediction as there exist systematic and concentration-dependent directed deviations of experimental and model data. However, from the results of this work, it is clear that there are different aspects for the prediction within a set of mixtures as well as for different sets of mixtures, which can be directional for the prediction of glass transition temperatures. In conclusion, this paper gives a deeper understanding of the glass transition behavior of anhydrous sugar mixtures as it opens up the view into mixtures with as little structural difference as possible and only the chain length of the mixing partners’ molecules was varied. It is therefore a small step in the direction of facing glass transition-related issues and to better evaluate and predict the storage stability of carbohydrate-rich systems.

## Figures and Tables

**Table 1:** List of the materials used in this study.

Material	Trade Name	Supplier
Glucose	D(+)-Glucose ≥ 99.5% CELLPURE^®^, water free	Carl Roth GmbH + Co. KG; Germany
Maltose	D(+)-Maltose Monohydrate ≥ 97%, CELLPURE^®^	Carl Roth GmbH + Co. KG; Germany
Maltotriose	Maltotriose 98% Powder	Thermo Fisher GmbH (Alfa Aesar); Germany
Maltotetraose	Maltotetraose DP4 (>99% HPLC)	ELICITYL Oligotech; France
Maltopentaose	Maltopentaose DP5 (>99% HPLC)	ELICITYL Oligotech; France
Maltohexaose	Maltohexaose DP6 (>99% HPLC)	ELICITYL Oligotech; France
Maltoheptaose	Maltoheptaose DP7 (>99% HPLC)	ELICITYL Oligotech; France

**Table 2:** Glass transition temperatures (*T_g_*) and changes in heat capacity at glass transition (Δ*c_p_*) for the pure components obtained by differential scanning calorimetry.

Carbohydrate	Monomer Count	Molecular Weight/g∗mol^−1^	Glass Transition *T_g_*/°C	Change in Specific Heat Capacity Δ*c_p_*/J∗g^−1^∗K^−1^
Glucose	1	180.16	38.87 ± 0.15	0.74 ± 0.02
Maltose	2	342.30	97.02 ± 0.58	0.53 ± 0.01
Maltotriose	3	504.44	130.18 ± 1.23	0.47 ± 0.01
Maltotetraose	4	666.58	153.13 ± 2.02	0.45 ± 0.16
Maltopentaose	5	828.72	172.91 ± 0.45	0.46 ± 0.04
Maltohexaose	6	990.86	178.53 ± 1.47	0.46 ± 0.08
Maltoheptaose	7	1153.00	188.44 ± 3.56	0.48 ± 0.04

## Data Availability

The data that support the findings of this study are available within the article and Appendix A. Further data are available from the corresponding author upon reasonable request.

## Appendix A

**Table A1:** Molar fractions (*x*), measured glass transition temperatures (*T_g_*), and glass transition temperatures according to the Gordon–Taylor (*T_gGT_*) and Couchman–Karasz equations (*T_gCK_*) of the ternary glucose mixtures. For the equimolar mixtures, the three glass transition values of the Gordon–Taylor equation correspond to fit of binary mixtures with varying glucose, maltose, maltotriose, and equimolar rest in this order.

xGlu/mol∗mol^−1^	xMal/mol∗mol^−1^	xMtr/mol∗mol^−1^	*T_g_*/°C	*T_g,GT_*/°C	*T_g,CK_*/°C	*T_g_*−*T_g,GT_*/°C	*T_g_*−*T_g,CK_*/°C
2/3	1/6	1/6	58.52 ± 0.68	59.44	57.08	−0.92	0.9
1/3	1/3	1/3	84.12 ± 0.30	83.32; 85.27; 84.34	80.61	0.79; −1.15; −0.22	3.08
1/6	2/3	1/6	91.07 ± 1.09	90.21	88.51	0.8	2.01
1/6	1/6	2/3	106.15 ± 2.03	105.95	102.33	0.20	3.97
